# Pancreatic Cancer Progression Is Regulated by IPO7/p53/LncRNA MALAT1/MiR-129-5p Positive Feedback Loop

**DOI:** 10.3389/fcell.2021.630262

**Published:** 2021-10-01

**Authors:** Jin Xu, Weixue Xu, Yang Xuan, Zhen Liu, Qinyun Sun, Cheng Lan

**Affiliations:** ^1^Department of Pancreatic and Thyroid Surgery, Shengjing Hospital, China Medical University, Shenyang, China; ^2^Department of Gastroenterology, Affiliated Hainan Hospital, Hainan Medical University, Hainan General Hospital, Hainan, China

**Keywords:** IPO7, p53, MALAT1, miR-129-5p, pancreatic cancer

## Abstract

**Background:** Pancreatic cancer is a malignancy with poor prognosis. Importin 7 (IPO7) is a soluble nuclear transport factor, which has been linked to the pathogenesis of several human diseases. However, its role and underlying mechanism in pancreatic cancer are still obscure.

**Methods:** Immunohistochemical staining and quantitative real-time polymerase chain reaction (qPCR) were performed to determine IPO7 expression in pancreatic cancer tissues and adjacent tissues. Western blot was used to measure IPO7 expression at the protein level in cell lines. Cell Counting Kit-8 (CCK-8), 5-bromo-2′-deoxyuridine (BrdU), flow cytometry, and Transwell assays were employed to explore the biological functions of IPO7. Subcutaneous xenograft transplanted tumor model and caudal vein injection model in mice were also established to validate the oncogenic role of IPO7. Western blot and qPCR were utilized to detect the regulatory function of IPO7 on p53 and metastasis-associated lung adenocarcinoma transcript 1 (MALAT1), respectively. Interaction between MALAT1 and miR-129-5p and interaction between miR-129-5p and IPO7 were verified by bioinformatics prediction, qPCR, dual-luciferase reporter gene experiment, RNA immunoprecipitation (RIP), and pull-down assay.

**Results:** Upregulation of IPO7 in pancreatic cancer tissues was associated with adverse prognosis of the patients with pancreatic cancer. Knocking down IPO7 remarkably suppressed cancer cell proliferation and metastasis, while it promoted apoptosis. Overexpression of IPO7 facilitated the malignant phenotypes of pancreatic cancer cells. Mechanistically, IPO7 could repress the expression of p53 and induce the expression of MALAT1 but reduce miR-129-5p expression. Furthermore, miR-129-5p was identified as a posttranscriptional regulator for IPO7, and its inhibition led to IPO7 overexpression in pancreatic cancer cells.

**Conclusion:** IPO7 is a novel oncogene for pancreatic cancer, and IPO7/p53/MALAT1/miR-129-5p positive feedback loop facilitates the progression of this deadly disease.

## Graphical Abstract

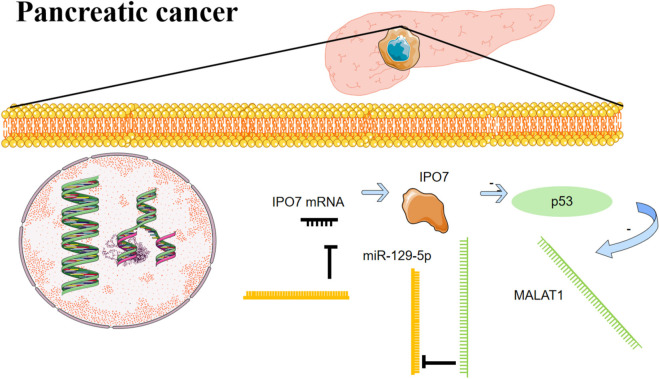

## Introduction

Pancreatic cancer is a highly fatal disease with a poor prognosis, and its main pathological type (more than 90% cases) is adenocarcinoma (Xu et al., 2020; Yin et al., 2020). Pancreatic cancer is considered one of the most deadly cancers, and its 5-year survival rate is only about 8% (Wang et al., 2018; Batista and Melo, 2019; Xu et al., 2020). Understanding the mechanism of pancreatic cancer progression is essential for developing effective therapeutic strategies for pancreatic cancer patients.

Importin-β family proteins are nucleocytoplasmic transport receptors, which are responsible for importing and exporting proteins and RNAs through the nuclear pores (Kimura and Imamoto, 2014). Importin 7 [IPO7, also named “RAN-binding protein 7” (RANBP7)] is a member of the importin-β family (Freedman and Yamamoto, 2004; Szczyrba et al., 2013). Previous studies have shown that IPO7 is significantly upregulated in a variety of tumors, such as prostate cancer and colorectal cancer, and glioblastoma multiforme (Li et al., 2000; Szczyrba et al., 2013; Xue et al., 2015). Besides, previous research shows that IPO7 depletion triggers the activation of p53, the famous tumor suppressor (Golomb et al., 2012). However, the role of IPO7/p53 axis in pancreatic cancer progression is unclear.

A growing body of evidence shows that long non-coding RNAs (lncRNAs) are implicated in multiple biological processes, including differentiation, proliferation, apoptosis, immune response, migration, and so on (Yu et al., 2020). LncRNAs’ abnormal expression is reported in multiple types of cancers, and they are associated with tumorigenesis and cancer progression (Tomar et al., 2020). Metastasis-associated lung adenocarcinoma transcript 1 (MALAT1) is transcribed from human chromosome 11q13 (Xiao et al., 2020). Reportedly, MALAT1 expression is significantly upregulated in lung cancer, glioblastoma, gastric cancer, as well as other tumors (Wei et al., 2019; Baspinar et al., 2020; Shao et al., 2020). MALAT1 is also reported to be overexpressed in pancreatic cancer, and MALAT1 knockdown repressed the malignant biological behaviors of pancreatic cancer cells (Cheng et al., 2018; Zhuo et al., 2018).

MicroRNAs (miRNAs), short non-coding RNAs that consist of 18–25 nucleotides, can interact with the 3′-untranslated region (3′-UTR) of messenger RNAs (mRNAs) and inhibit translation (Lu et al., 2020; Tamaru et al., 2020). MiR-129-5p suppresses the malignant phenotypes of multiple cancers (Lin et al., 2019; Qiu et al., 2019; He et al., 2020; Wan et al., 2020). However, the mechanism causing the dysregulation of miR-129-5p in cancer biology remains to be further explored.

The present research revealed that IPO7 was significantly upregulated in pancreatic cancer, and it markedly facilitated the proliferation, migration, and invasion of pancreatic cancer cells. Importantly, we found that there was an IPO7/p53/MALAT1/miR-129-5p positive feedback loop in pancreatic cancer progression. This work helps clarify the mechanism of sustaining progression of pancreatic cancer.

## Materials and Methods

### Human Samples

Fifty-five pairs of pancreatic cancer tissues and corresponding normal tissues (at >1 cm from the edge of the primary tumor) were obtained from the patients during surgery at Shengjing Hospital between 2017 and 2018. Each patient signed the informed consent before the surgery. All tissue specimens were collected, immediately snap-frozen using liquid nitrogen, and stored at -80°C until RNA extraction. This research was endorsed by the ethics committee of Shengjing Hospital and performed according to the Declaration of Helsinki.

### Cell Lines and Cell Culture

Normal human pancreatic duct epithelial cell line (HPDE6-C7 cell) and five different human pancreatic cancer cell lines, including PANC-1, HS_766T, BxPC-3, AsPC-1, and HPAC cells, were purchased from the China Center for Type Culture Collection (CCTCC, Wuhan, China). Dulbecco’s modified Eagle’s medium (DMEM, Thermo Fisher Scientific, Rockville, MD, United States) containing 10% fetal bovine serum (FBS) (Hyclone, South Logan, UT, United States), 100 U/ml penicillin, and 0.1 mg/ml streptomycin (Hyclone, South Logan, UT, United States) were employed to culture the cells in a humidified incubator at 37°C in 5% CO_2_.

### Cell Transfection

Small interfering RNA (siRNA) targeting IPO7 and MALAT1 (si-IPO7 and si-MALAT1), control siRNA (si-NC), miR-129-5p mimics, miR-129-5p inhibitors, pcDNA3.1 vectors overexpressing IPO7 and MALAT1, and the corresponding miRNA control and empty vector were synthesized and provided by GenePharma Co., Ltd. (Guangzhou, China). Vectors and siRNAs (50 nmol) and miRNA mimics or inhibitors (20 nmol) were transfected into pancreatic cancer cells using Lipofectamine^®^ 2000 (Invitrogen, Carlsbad, CA, United States) according to the manual. After transfection for 48 h, quantitative polymerase chain reaction (qPCR) and Western blot were performed to measure the transfection efficiency.

### RNA Extraction and Quantitative Polymerase Chain Reaction

Total RNA was extracted from tissue samples or cell lines using the TRIzol reagent (Life Technologies Corporation, Carlsbad, CA, United States) under the guidance of the protocols. The extracted RNA was reversely transcribed into complementary DNA (cDNA) using a High-Capacity cDNA Reverse Transcription Kit (Applied Biosystems, Foster, CA, United States). qPCR was executed using SYBR^®^ Premix Ex Taq^TM^ II (TaKaRa, Dalian, China) on ABI 7500 Fast Real-Time PCR System (Applied Biosystems, Foster City, CA, United States). Glyceraldehyde 3-phosphate dehydrogenase (GAPDH) and U6 were used as endogenous controls. The fold change of the gene expression levels was calculated by the 2^–ΔΔCt^ method. The sequences of the primers used in this work were as follows:

IPO7: forward, 5′-TGGGACCTGATCATGCAACC-3′; reverse, 5′-AGCTGCCTTCATGACATCCC-3′
MALAT1: forward, 5′-GCCTGGAAGCTGAAAAACGG-3′; reverse, 5′-TGGAAAACGCCTCAATCCCA-3′
p53: forward, 5′-GAGGTTGGCTCTGACTGTACC-3′; reverse, 5′-TCCGTCCCAGTAGATTACCAC-3′
GAPDH: forward, 5′-CATGAGAAGTATGACAACAGCCT-3′; reverse, 5′-AGT CCTTCCACGATACCAAAGT-3′
U6: forward, 5′-GCTTCGGCAGCACATATACT-3′; reverse, 5′-GCAGGGTCCGAGGTATTC-3′.

### Immunohistochemistry

Immunohistochemistry (IHC) was executed to determine IPO7 protein expression in 55 pairs of pancreatic cancer tissues and adjacent tissues. The specimens were fixed in 10% formaldehyde and embedded in paraffin. Next, the paraffin block was sliced, and the sections were dewaxed and rehydrated. Antigen retrieval was performed in a microwave by placing the sections in epitope retrieval solution (0.01 M citrate buffer, pH 6.0) for 5 min; endogenous peroxidase was inhibited by immersing the sections in 0.3% hydrogen peroxide for 10 min. Then, the sections were blocked in 5% bovine serum for 30 min. Then, anti-IPO7 antibody (ab99273, Abcam, 1:100) was supplemented to incubate the sections at 4°C for 12 h in a humidified box. Subsequently, the sections were rinsed with phosphate buffered saline (PBS) and then incubated with a biotin-linked antiserum for 1 h. Next, the sections were rinsed again and stained with 3,3-diaminobenzidine. Finally, the sections were observed under a microscope, and the staining was examined by two independent pathologists.

### Cell Viability Assay

The cells in each group (2 × 10^3^ cells/well) were cultured in 96-well plates for different times (24, 48, 72, and 96 h). At each time point, 10 μl of Cell Counting Kit-8 (CCK-8) solution (Dojindo, Tokyo, Japan) was supplemented into each well and incubated for 2 h. Next, the absorbance of each well at 450 nm was measured by a microplate reader (Model 550; Bio-Rad Laboratories, Inc., Hercules, CA, United States). The cell proliferation curve of the cells in each group was plotted 96 h later using time as the abscissa and absorbance as the ordinate.

### Flow Cytometry Analysis

Apoptosis of pancreatic cancer cells was monitored using an Annexin V–fluorescein isothiocyanate (FITC)/propidium iodide (PI) Apoptosis Detection Kit (BD Biosciences, San Jose, CA, United States). The density of the cells in each sample was adjusted after they were rinsed twice with PBS. The cells were then resuspended in 400 μl of binding buffer, thoroughly mixed with 5 ml of Annexin V–FITC staining solution and 5 ml of PI staining solution, and then incubated at room temperature for 15 min in the dark. Following that, a flow cytometer (AttuneNxT; Thermo Fisher Scientific, Waltham, MA, United States) was utilized to monitor cell apoptosis within 1 h.

### Cell Migration and Invasion Experiments

Transwell assay was conducted using Transwell chambers (pore size, 8 μM; Corning, NY, United States) (for the migration assay) or Transwell chambers pre-coated with Matrigel (1:10; BD Biosciences, Franklin Lakes, NJ, United States) (for the invasion assay). Briefly, 200 μl of serum-free medium containing 5 × 10^4^ cells were seeded into the upper chamber of each well, and 700 μl of medium containing 20% FBS was added to the lower chamber. Following that, the cells were cultured for 24 h. Subsequently, the pancreatic cancer cells remaining on the upper side of the membrane were scraped off, and the rest of the cells were fixed with 4% paraformaldehyde for 15 min and stained with 0.1% crystal violet solution for 30 min. Ultimately, a Leica DM4000B microscope (Leica, Wetzlar, Germany) was employed to count the number of migrated or invaded cells in three fields of view, and then the average was calculated.

### Western Blot

Extraction of total protein was performed with radioimmunoprecipitation assay (RIPA) lysis buffer (Beyotime, Shanghai, China). Then, the protein was quantified using the bicinchoninic acid (BCA) Protein Assay Kit (Thermo Fisher Scientific, Waltham, MA, United States). Following that, the proteins were mixed with loading buffer, heated in boiling water for 10 min for denaturation, dissolved by sodium dodecyl sulfate–polyacrylamide gel electrophoresis, and then transferred onto polyvinylidene fluoride (PVDF) membrane (Millipore, Billerica, MA, United States). After blocking with 5% skimmed milk for 1 h, the membranes were incubated with primary antibodies [anti-IPO7 antibody, ab99273, Abcam, 1:500; anti-E-cadherin antibody, ab231303, Abcam, 1:500; anti-N-cadherin antibody, ab76011, Abcam, 1:500; anti-N-cadherin antibody, ab92547, Abcam, 1:500; anti-Snail antibody, ab216347, Abcam, 1:500; anti-alpha-smooth muscle actin (aSMA) antibody, ab184705 Abcam, 1:500; anti-GAPDH antibody, ab8245, Abcam, 1:2,000] overnight at 4°C. The membrane was rinsed and then incubated with horseradish peroxidase (HRP)-conjugated secondary antibody (Beyotime, Shanghai, China) for 2 h at room temperature. Ultimately, the protein bands were developed with an enhanced chemiluminescence (ECL) kit (Biossci, Wuhan, China).

### *In vivo* Experiment

The animal experiments were endorsed by the Experimental Animal Ethics Committee of Shengjing Hospital. Ten BALB/c athymic nude mice (aged 4 weeks, five mice in each group) were injected subcutaneously with 2 × 10^7^ HPAC cells. All mice were housed in a pathogen-free facility with a temperature of 24°C ± 1°C, a humidity of 50%, 12-h light–dark cycle, and *ad libitum* access to water and food. Tumor volume (length × width^2^ × 0.5) was measured every 1 week. In the lung metastasis experiment, 1 × 10^7^ HPAC cells were injected into the caudal vein of each mouse (five mice in each group). The nude mice were sacrificed after 14 days, and lung colonization was measured using H&E staining and pathological analysis.

### Dual-Luciferase Reporter Gene Experiment

The reporter plasmids [psiCHECK2 vector containing the wild-type (WT)-MALAT1 sequence or mutant (MUT) sequence and psiCHECK2 vector containing WT IPO7 sequence or MUT sequence] were constructed by Promega (Madison, WI, United States). The reporter plasmids were co-transfected with miR-129-5p mimic or control miRNAs into pancreatic cancer cells, and then the cells were cultured for 24 h. Next, the cells were harvested, and the luciferase activity of each group was examined by a dual-luciferase reporter assay system (Promega, Madison, WI, United States). Firefly luciferase activity was normalized to Renilla luciferase activity.

### RNA Immunoprecipitation Experiment

RNA immunoprecipitation (RIP) experiments were performed with Magna RIP RNA-Binding Protein Immunoprecipitation Kit (Millipore, Billerica, MA, United States). Briefly, pancreatic cancer cells were lysed with RIP lysis buffer, and 100 μl of lysate were incubated with magnetic beads pre-conjugated with anti-Argonaute 2 (anti-Ago2, 1:50, cat. no. ab186733, Abcam) or immunoglobulin G (IgG) antibody (anti-IgG, 1:100, cat. no. ab48386, Abcam) at 4°C for 6 h. Subsequently, the samples were incubated with proteinase K to eliminate the proteins. After isolation of the immunoprecipitated RNA, qPCR was performed to detect the enrichment of miR-129-5p and MALAT1.

### RNA Pull-Down Assay

The RNA pull-down experiments were conducted to evaluate the interaction between MALAT1 and miR-129-5p. In short, the pancreatic cancer cells were transfected with biotin-labeled miR-129-5p or biotin-labeled control miRNAs. Next, the cells were lysed in lysis buffer, and the mixtures were incubated on ice for 5 min. After centrifugation, the supernatant was collected and incubated at 4°C for 2 h with magnetic beads (Genescript, Nanjing, China), and the RNA was isolated using the RNeasy Mini Kit (QIAGEN, Duesseldorf, Germany). Ultimately, MALAT1 expression in the pull-down compounds of biotin-miR-129-5p was detected by qPCR.

### Statistical Analyses

The experiments were repeated at least three times. The data were analyzed by SPSS 20.0 software (Abbott Laboratories, Chicago, IL, United States), and the figures were plotted by GraphPad Prism 8.0 (GraphPad Software, Inc., La Jolla, CA, United States). All data were presented as “mean value ± standard deviation (SD).” Kolmogorov–Smirnov test was used to determine the normality of the distribution of data in each group. Student’s *t*-test, Wilcoxon signed-rank test, and one-way ANOVA were used for comparing the differences among groups.

## Results

### Importin 7 Expression Was Elevated in Pancreatic Cancer Tissues and Linked to Adverse Prognosis

**FIGURE 1:**
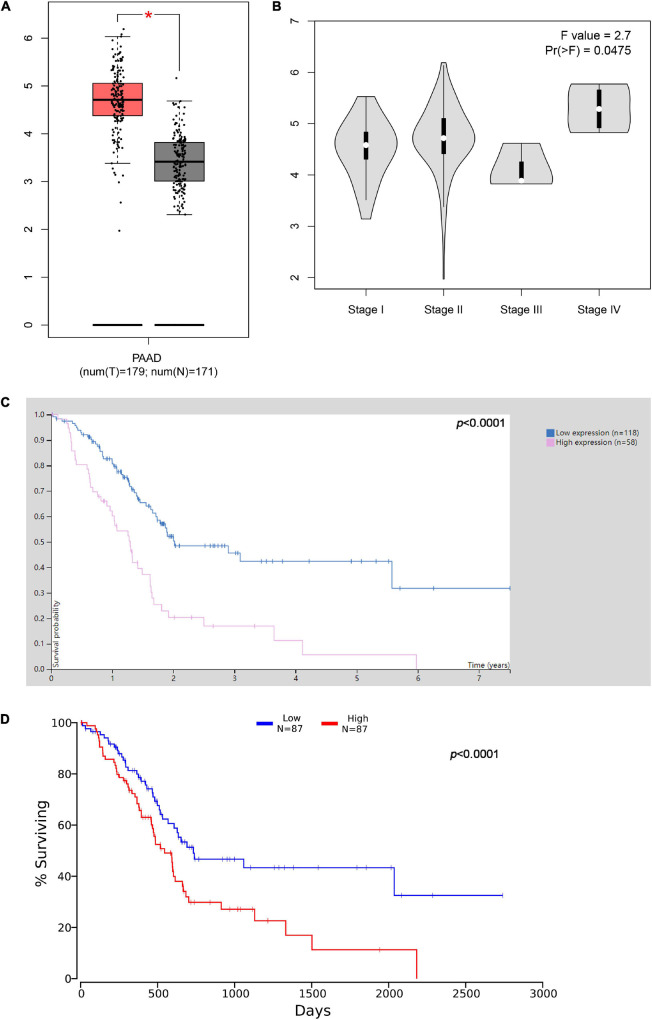
The expression level of importin 7 (IPO7) in pancreatic adenocarcinoma (PAAD) and its significance. **(A)** The expression of IPO7 in pancreatic cancer tissues in The Cancer Genome Atlas (TCGA) database was analyzed through Gene Expression Profiling Interactive Analysis (GEPIA) database (http://gepia2.cancer-pku.cn/#index). **(B)** The expression levels of IPO7 in pancreatic cancer tissues of different clinical stages were analyzed *via* GEPIA database. **(C,D)** Human Protein Atlas database (http://www.proteinatlas.org/) and Oncolnc database (www.oncolnc.org) indicated that high expression of IPO7 was associated with shorter survival time of patients with pancreatic cancer *p < 0.05.

**FIGURE 2:**
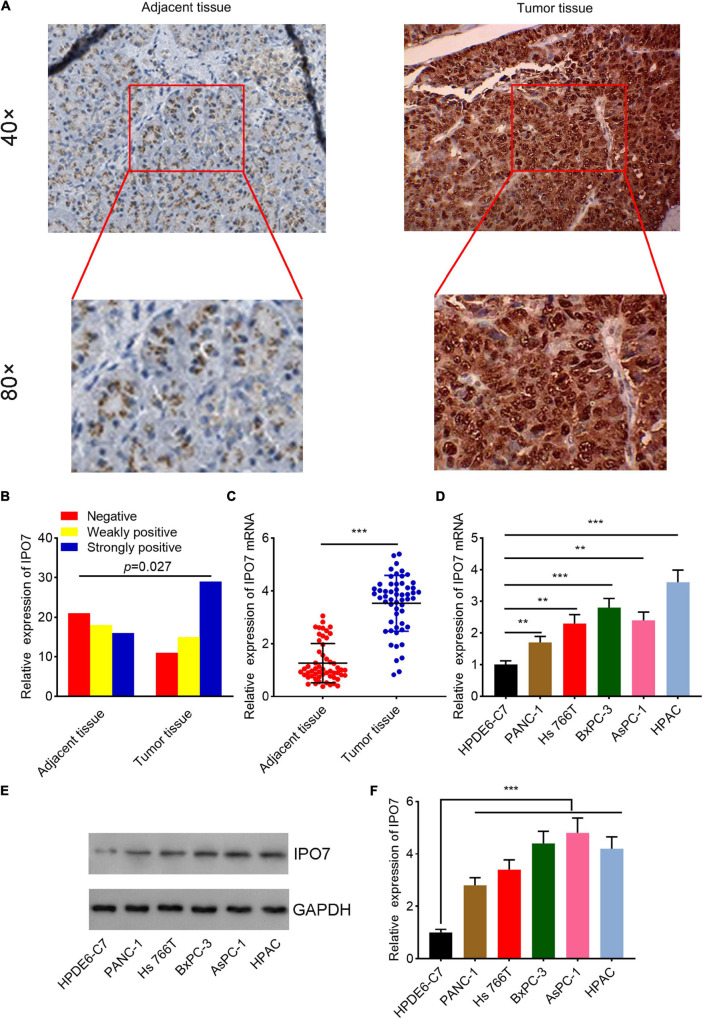
Importin 7 (IPO7) is upregulated in pancreatic cancer tissue and cells. **(A)** Representative immunohistochemical images of pancreatic cancer tissues and adjacent tissues. **(B)** The strong positive rate, weak positive rate, and negative rate of IPO7 expression in 55 pairs of pancreatic cancer and adjacent tissues were compared. **(C)** qPCR was performed to evaluate the expression of IPO7 mRNA in pancreatic cancer tissues and adjacent tissues. **(D–F)** qPCR **(D)** and Western blot **(E,F)** were performed to determine the expression of IPO7 in normal human pancreatic duct epithelial cells (HPDE6-C7) and five different human pancreatic cancer cell lines (PANC-1, HS_766T, BxPC-3, AsPC-1, and HPAC) at the mRNA level and protein level. **p < 0.01, ***p < 0.001.

By analyzing The Cancer Genome Atlas (TCGA) data through Gene Expression Profiling Interactive Analysis (GEPIA)^1^ database, it was revealed that IPO7 was remarkably overexpressed in pancreatic adenocarcinoma (PAAD) specimens (Figure 1A). Furthermore, we found that IPO7 expression was even higher in patients with stage IV PAAD (Figure 1B). Besides, survival analysis was performed by two databases, including the Human Protein Atlas database^2^ and Oncolnc database.^3^ The results suggested that the prognosis of patients with IPO7 high expression was worse than that of the patients with low IPO7 expression (Figures 1C,D), indicating that it may be a potential prognostic biomarker of pancreatic cancer. Additionally, we detected IPO7 expression in carcinoma tissues and adjacent tissues of 55 patients with pancreatic cancer by IHC (Figure 2A). The statistical results showed that IPO7 positive rate in pancreatic cancer tissues was markedly higher than that in the adjacent tissues (Figure 2B). Besides, qPCR and Western blot suggested that IPO7 expression was remarkably enhanced in pancreatic cancer tissues and cell lines at mRNA and protein levels compared with normal tissues and cells (Figures 2C–F).

### Importin 7 Enhanced Pancreatic Cancer Cell Proliferation, Migration, Invasion, and Restrained Cell Apoptosis

**FIGURE 3:**
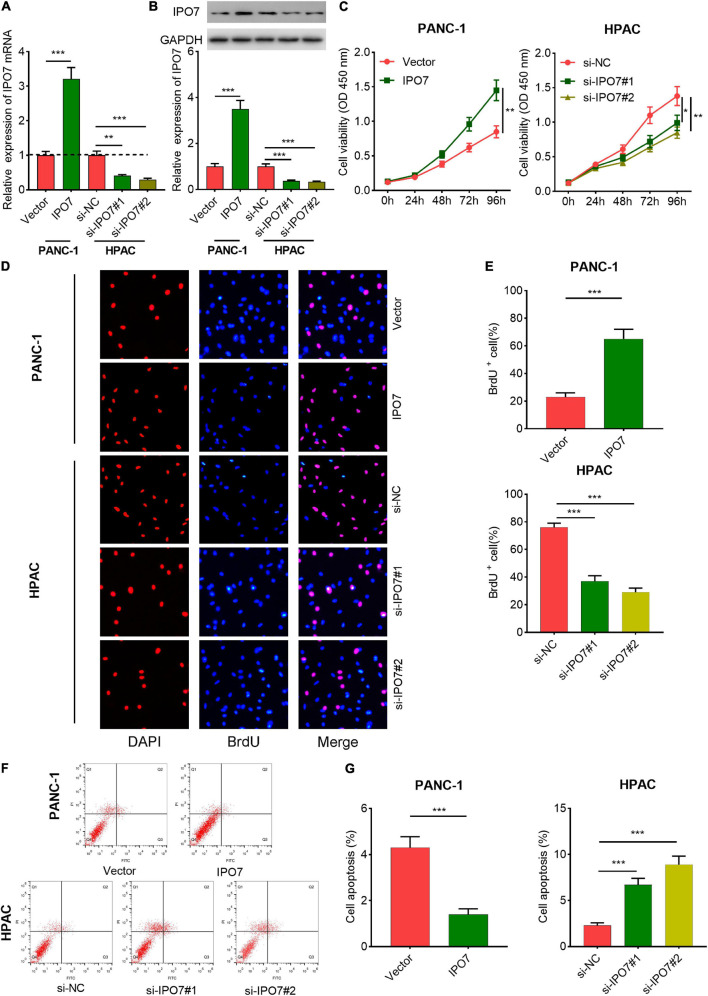
Importin 7 (IPO7) regulates pancreatic cancer cell proliferation and inhibits cell apoptosis. **(A,B)** qPCR and Western blot indicated that a cell model with overexpression of IPO7 and a cell model with IPO7 depletion were successfully constructed. **(C–E)** Cell Counting Kit-8 (CCK-8) and 5-bromo-2′-deoxyuridine (BrdU) assays were used to assess the proliferation of pancreatic cancer cells with IPO7 overexpression or knockdown. **(F,G)** Flow cytometry was performed to detect the apoptosis of pancreatic cancer cells with IPO7 overexpression or knockdown. *p < 0.01, **p < 0.01, ***p < 0.001.

**FIGURE 4:**
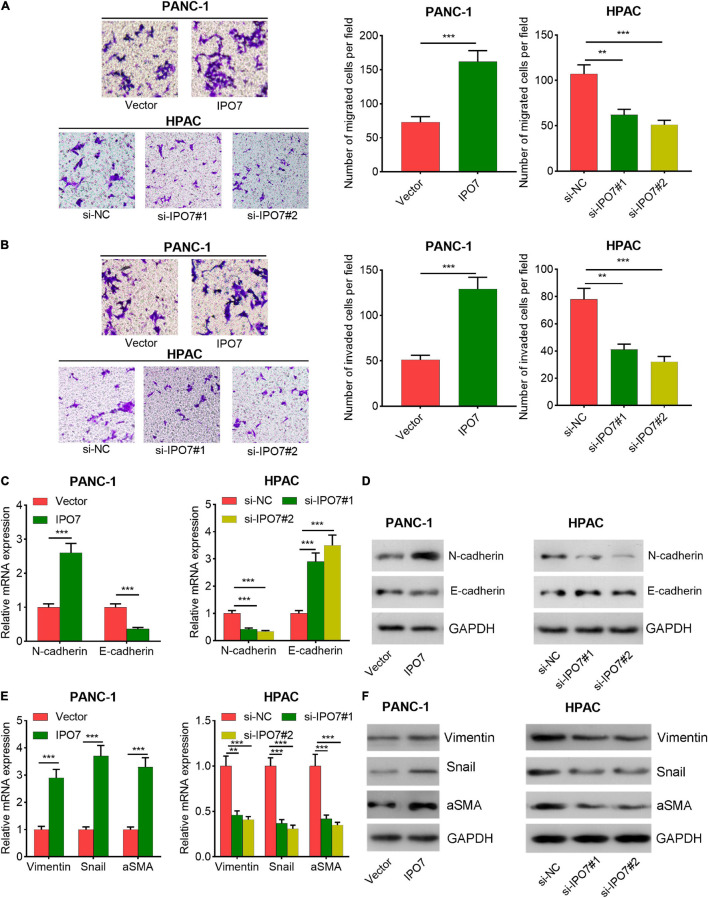
Importin 7 (IPO7) regulates pancreatic cancer cell migration and invasion. **(A,B)** The migration and invasion of pancreatic cancer cells were assessed by Transwell assay. **(C–F)** qPCR and Western blot were performed to determine the expression of epithelial–mesenchymal transition (EMT)-related indicators, including E-cadherin, N-cadherin, vimentin, Snail, and alpha-smooth muscle actin (aSMA). **p < 0.01, ***p < 0.001.

Subsequently, IPO7 overexpression plasmid was transfected into PANC-1 cells, and two different siRNAs against IPO7 were transfected into HPAC cells (Supplementary Figure 1 and Figures 3A,B). CCK-8 and 5-bromo-2′-deoxyuridine (BrdU) assays indicated that IPO7 overexpression had a promoting effect on the proliferation of PANC-1 cells compared with the control group (Figures 3C–E). Furthermore, the results of flow cytometry showed that the apoptotic rate was downregulated in PANC-1 cells with IPO7 overexpression (Figures 3F,G). Transwell assay implied that IPO7 upregulation significantly contributed to the migration and invasion of PANC-1 cell (Figures 4A,B). Besides, the results of qPCR and Western blot indicated that N-cadherin, vimentin, Snail, and aSMA were increased after IPO7 overexpression, whereas the expression of E-cadherin was repressed by IPO7, suggesting that IPO7 promoted the epithelial–mesenchymal transition (EMT) of pancreatic cancer cells (Figures 4C–F). Conversely, the depletion of IPO7 restrained cell proliferation, migration, invasion, and EMT process and induced cell apoptosis (Figures 3C–G, 4A–F). Collectively, these data suggested that IPO7 promoted pancreatic cancer progression.

### Importin 7 Negatively Regulated p53 and Positively Regulated the Expression of MALAT1

**FIGURE 5:**
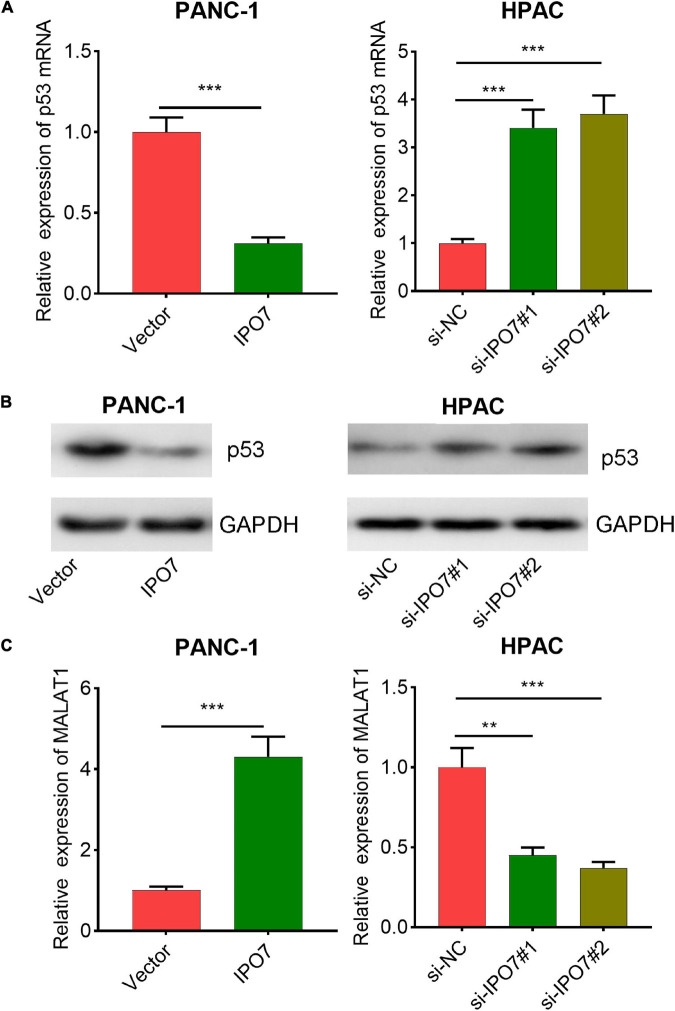
p53 and metastasis-associated lung adenocarcinoma transcript 1 (MALAT1) are regulated by importin 7 (IPO7). **(A)** The expression of p53 mRNA in PANC-1 cells with IPO7 overexpression and HPAC cells with IPO7 knockdown was detected by qPCR. **(B)** The expression of p53 in PANC-1 cells with IPO7 overexpression and HPAC cells with IPO7 knockdown was detected by Western blot. **(C)** The expression of MALAT1 in PANC-1 cells with IPO7 overexpression and HPAC cells with IPO7 knockdown was detected by qPCR. **p < 0.01, ***p < 0.001.

It is reported that IPO7 negatively regulates the expression of p53, the famous tumor suppressor (Golomb et al., 2012). Consistently, qPCR and Western blot revealed that IPO7 overexpression induced a decrease in p53 levels in PANC-1 cells, while knockdown of IPO7 induced an upregulation of p53 in HPAC cells (Figures 5A,B). Furthermore, p53 is also the inhibitory transcription factor of MALAT1, a well-known cancer-promoting lncRNA (Fuschi et al., 2017; Wang et al., 2019). In the present work, our data suggested that IPO7 positively regulated MALAT1 expression in pancreatic cancer (Figure 5C). In addition, compared with p53 + MALAT1 group, the expression of MALAT1 in HAPC cells in p53 + MALAT1 + miR-129-5p group was decreased (Supplementary Figure 2A); this change could be partially reversed by IPO7 overexpression plasmid (Supplementary Figure 2A). On the other hand, compared with the si-NC group, the expression level of MALAT1 in PANC-1 cells was increased after inhibition of p53 (Supplementary Figure 2B). Furthermore, compared with the si-p53 + si-MALAT1 group, the expression of MALAT1 in si-p53 + si-MALAT1 + miR-129-5p inhibitors group was increased, and this effect could be partially reversed by IPO7 knockdown (Supplementary Figure 2B). These data suggested that IPO7/p53 could regulate MALAT1 expression in pancreatic cancer cells that was dependent on miR-129-5p.

### MALAT1 Worked as a CeRNA by Sequestering MiR-129-5p

**FIGURE 6:**
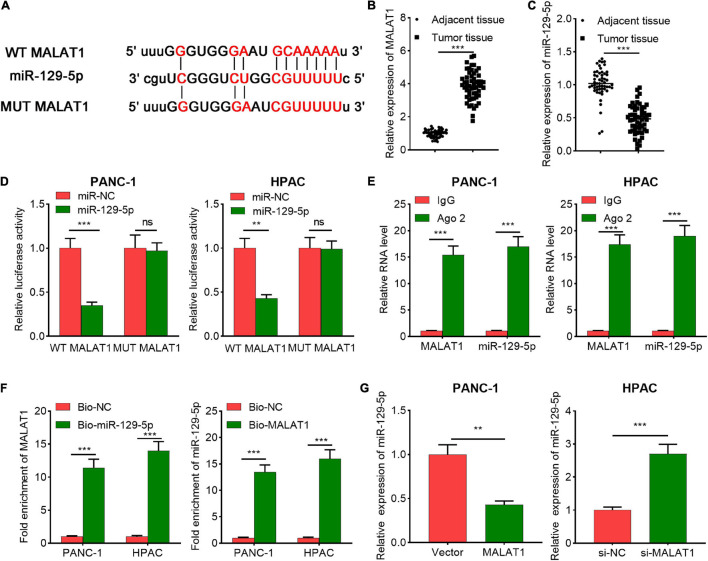
The relationship between metastasis-associated lung adenocarcinoma transcript 1 (MALAT1) and miR-129-5p. **(A)** The predicted binding sequence between miR-129-5p and wide-type (WT) MALAT1 and the sequence of the mutant type (MUT) of MALAT1. **(B)** The expression of MALAT1 in pancreatic cancer tissue and adjacent tissue was detected by qPCR. **(C)** The expression level of miR-129-5p in pancreatic cancer tissue and adjacent tissue was measured by qPCR. **(D)** The binding site between miR-129-5p and MALAT1 was verified by dual-luciferase reporter assay. **(E)** RNA immunoprecipitation (RIP) assay was performed with immunoglobulin g (IgG) or Ago2 antibody in pancreatic cancer cells, and the co-precipitated RNA was used to perform qPCR for MALAT1 and miR-129-5p detection. **(F)** RNA pull-down assay confirmed that MALAT1 interacted with miR-129-5p. Relative enrichment of MALAT1 and miR-129-5p was measured using qPCR. **(G)** qPCR showed that MALAT1 could negatively regulate miR-129-5p expression in pancreatic cancer cells. **p < 0.01, ***p < 0.001.

For further expounding the molecular mechanism of IPO7 in regulating pancreatic cancer progression, StarBase and DIANA LncBase Predicted v.2 were performed to explore the miRNAs that potentially interacted with MALAT1, and miR-129-5p was among the candidate miRNAs (Figure 6A). Compared with adjacent tissue, the expression level of MALAT1 was significantly increased and the expression level of miR-129-5p was significantly decreased in pancreatic cancer tissues (Figures 6B,C). To further investigate the regulatory relationship between MALAT1 and miR-129-5p, dual-luciferase reporter gene experiment was performed, and it was revealed that transfection of miR-129-5p mimics significantly reduced the luciferase activity of WT-MALAT1 reporter plasmid, while transfection of miR-129-5p mimics did not change the luciferase activity of MUT-MALAT1 reporter plasmid (Figure 6D). Furthermore, RIP experiments confirmed that MALAT1 and miR-129-5p were enriched in Ago2-containing microribonucleoproteins compared with IgG group (Figure 6E). Consistently, RNA pull-down experiment illustrated that MALAT1 was enriched in the compounds containing biotin-miR-129-5p (Figure 6F). Besides, MALAT1 negatively modulated miR-129-5p expression in pancreatic cancer cells (Figure 6G). Collectively, our results indicated that miR-129-5p was a downstream target of MALAT1 in pancreatic cancer cells.

### MiR-129-5p Directly Targeted Importin 7

**FIGURE 7:**
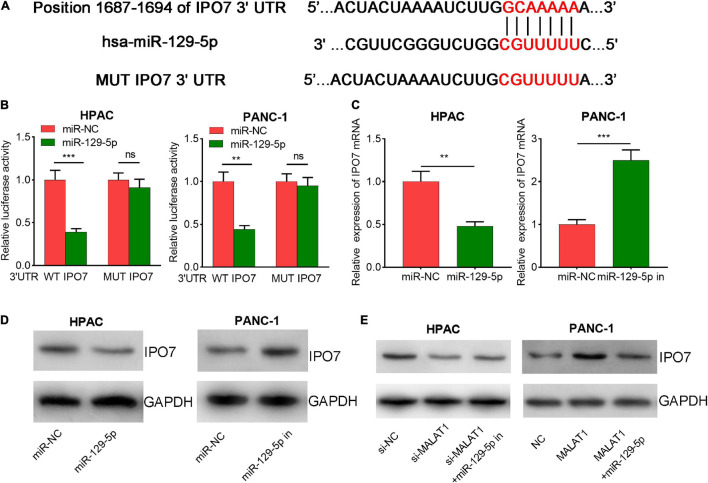
Importin 7 (IPO7) is a direct target of miR-129-5p in pancreatic cancer cells. **(A)** The wild-type (WT) binding site between IPO7 3′-untranslated region (UTR) and miR-129-5p was shown, and the mutant (MUT) sequence was designed. **(B)** miR-129-5p mimics significantly suppressed the luciferase activity of reporter vector carrying wild-type (WT) IPO7 3′-UTR sequence, but not MUT IPO7 3′-UTR sequence. **(C,D)** qPCR and Western blot showed that miR-129-5p could negatively regulate IPO7 expression in pancreatic cancer cells. **(E)** Western blot showed that the positive regulation of IPO7 expression by MALAT1 could be partially weakened by miR-129-5p. *p < 0.05, **p < 0.01, ***p < 0.001.

Next, bioinformatics analysis was performed with StarBase, TargetScan, and miRDB databases, and interestingly, IPO7 was predicted as a potential downstream target for miR-129-5p (Figure 7A and Supplementary Figure 3). Then, we performed Kyoto Encyclopedia of Genes and Genomes (KEGG) analysis to predict the potential biological functions of miR-129-5p integrated signature. The results showed that the targets of miR-129-5p were mainly associated with multiple cancer-related pathways, including the Hippo, WNT, and p53 signaling pathways (Supplementary Figure 4). The dual-luciferase reporter gene experiment suggested that the transfection of miR-129-5p mimics significantly decreased the luciferase activity of WT-IPO7 3′-UTR reporter plasmid, while the transfection of miR-129-5p mimics did not change the luciferase activity of MUT IPO7 3′-UTR reporter plasmid (Figure 7B). Moreover, qPCR and Western blot suggested that the transfection of miR-129-5p mimics markedly repressed IPO7 expression, but inhibition of miR-129-5p increased IPO7 expression (Figures 7C,D). Besides, MALAT1 overexpression also promoted IPO7 expression, and this process could be partially reversed by the co-transfection of miR-129-5p mimics (Figure 7E). Briefly, the findings indicated that IPO7 was a downstream target of miR-129-5p and could be positively regulated by MALAT1.

### Correlations Were Observed Among the Expressions of Importin 7, p53, MALAT1, and MiR-129-5p in Pancreatic Cancer

**FIGURE 8:**
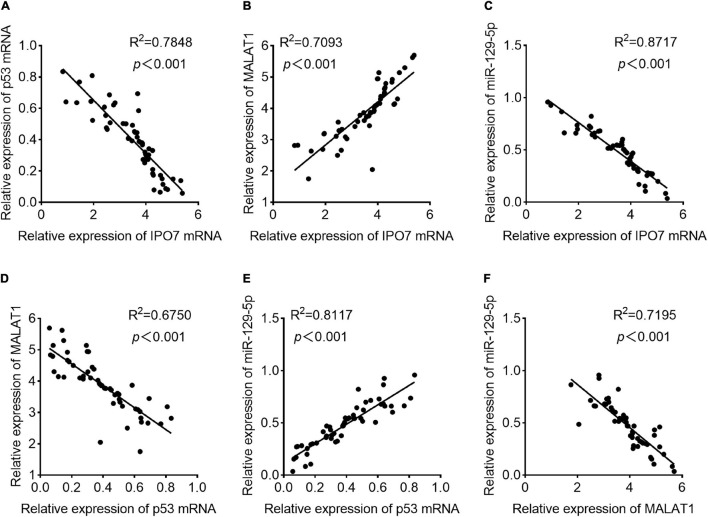
The correlations among the expression levels of importin 7 (IPO7), p53, metastasis-associated lung adenocarcinoma transcript 1 (MALAT1), and miR-129-5p in pancreatic cancer tissues. **(A)** The expression level of IPO7 mRNA was negatively correlated with that of p53 mRNA. **(B)** The expression level of IPO7 mRNA was positively correlated with the expression level of MALAT1. **(C)** The expression level of IPO7 mRNA was negatively correlated with the expression level of miR-129-5p. **(D)** The expression level of p53 mRNA was negatively correlated with that of MALAT1. **(E)** The expression level of p53 mRNA was positively correlated with the expression level of miR-129-5p. **(F)** The expression level of MALAT1 was negatively correlated with the expression level of miR-129-5p.

Subsequently, in pancreatic cancer samples, Pearson’s correlation analysis indicated that IPO7 expression was negatively correlated with p53 expression and miR-129-5p expression and positively correlated with MALAT1 expression (Figures 8A–C). Additionally, p53 expression and MALAT1 expression were negatively correlated, and p53 expression was positively correlated with miR-129-5p expression (Figures 8D,E). Besides, MALAT1 and miR-129-5p expressions were found to be negatively correlated in pancreatic cancer samples (Figure 8F). Based on the results mentioned above, it was concluded that there existed an IPO7/p53/MALAT1/miR-129-5p positive feedback loop in pancreatic cancer progression.

### Inhibition of Importin 7 Suppressed Tumor Growth and Metastasis *in vivo*

**FIGURE 9:**
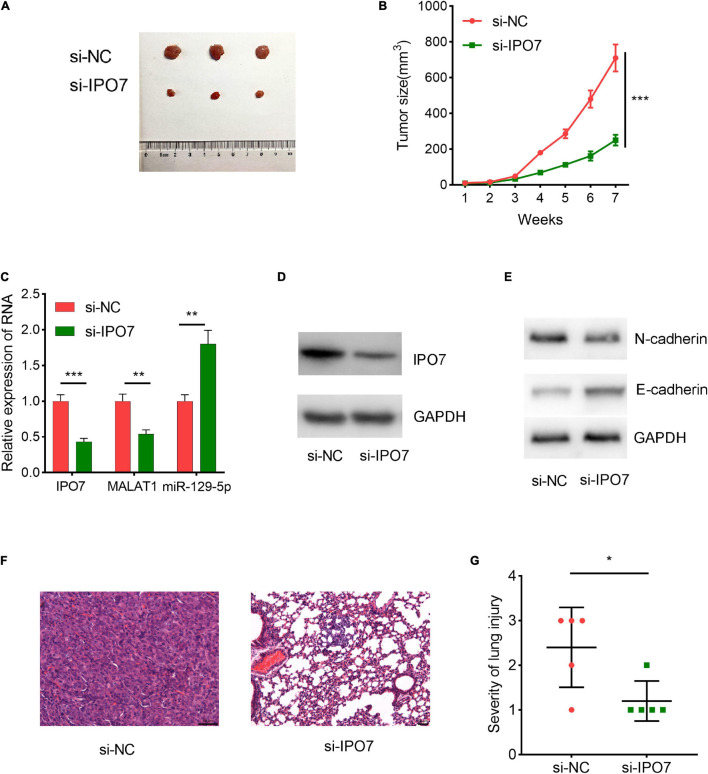
Importin 7 (IPO7) knockdown can restrain the growth and metastasis of pancreatic cancer cells *in vivo*. **(A,B)** IPO7 depletion reduced the tumor size *in vivo.* **(C)** The expression of IPO7, metastasis-associated lung adenocarcinoma transcript 1 (MALAT1), and miR-129-5p in tumor tissues of the mice was determined by qPCR. **(D,E)** The expression of E-cadherin and N-cadherin in tumor tissues of the mice was determined by Western blot. **(F)** Representative hematoxylin and eosin staining images of the lung metastasis of the mice. **(G)** Chi-square test was used to analyze the difference in the degree of lung metastasis between the two groups (3, severe; 2, moderate; 1, mild). *p < 0.05, **p < 0.01, ***p < 0.001.

Next, we performed animal experiments to further validate the biological effects of IPO7. HPAC cells with stable IPO7 knockdown and the cells in the control group were inoculated into nude mice, respectively, and the tumor growth was measured. It was observed that the average tumor size in the IPO7 knockdown group was significantly lower compared with that in the control group (Figures 9A,B). Additionally, IPO7 knockdown reduced the expression of MALAT1 and N-cadherin in the tumor tissues but increased the expression of miR-129-5p and E-cadherin (Figures 9C–E). Additionally, hematoxylin and eosin staining indicated that the knockdown of IPO7 reduced lung metastasis of pancreatic cancer cells (Figures 9F,G). These results further validated that IPO7 regulated the proliferation and metastasis of pancreatic cancer cells.

## Discussion

Further clarification of the mechanism of pancreatic cancer progression will help improve the diagnosis and treatment of the patients with this deadly disease (Dardare et al., 2020). Importins contain a Ran-GTP binding domain that mediates RanGTPase-dependent transport across the nuclear envelope (Hakim et al., 2013). IPO7, whose gene is located on chromosome 11, belongs to the Ran-binding protein superfamily and has been reported to work as a nuclear transport regulator (Szczyrba et al., 2013). IPO7 has been confirmed to heterodimerize with another importin subunit, Importin 3 (IPO3), and the formation of this heterodimer enables the translocation of the target proteins (Mohammad et al., 2016). Notably, IPO7 is frequently overexpressed in cancers, and it facilitates the progression of multiple types of cancers. For instance, it is reported that IPO7, together with importin 4 (IPO4), can regulate the nuclear localization of hypoxia-inducible factor (HIF)-1α, which is well-known as an oncoprotein with transcription factor activity (Chachami et al., 2009). Additionally, the translocation of mothers against decapentaplegic homolog (SMAD) family members, a class of proteins that are crucial regulators in cancer biology, from the cytoplasm to the nucleus, is also regulated by IPO7 (Yao et al., 2008). Importantly, it is reported that IPO7 depletion triggers p53 activation and p53-dependent cell cycle arrest; activation of p53 by IPO7 knockdown has distinct features of ribosomal biogenesis stress, with increased binding of Mdm2 to ribosomal proteins L5 and L11 (RPL5 and RPL11, respectively) (Golomb et al., 2012). In the present study, we found that IPO7 expression in pancreatic cancer tissues was markedly elevated, and its overexpression was significantly associated with shorter survival of pancreatic cancer patients. Moreover, functional experiments confirmed that overexpression of IPO7 significantly promoted the proliferation, migration, invasion, and inhibition of apoptosis of pancreatic cancer cells, accompanied by decreased expression of p53. Meanwhile, IPO7 knockdown could repress the malignant features of pancreatic cancer cells, accompanied by the increased expression of p53. For the first time, the present work proved that IPO7 was a novel oncogene in pancreatic cancer, which exerted its biological function partly *via* repressing p53, and these results suggested that targeting IPO7 could probably be a promising strategy to treat pancreatic cancer.

It is worth noting that, in recent years, accumulating studies have confirmed that lncRNAs exert a vital regulatory effect in the development of diverse malignancies (Zhang et al., 2020). MALAT1 is well-known as an oncogenic lncRNA in multiple cancers. For instance, MALAT1 facilitates the development of gastric cancer through activation of the phosphoinositide 3-kinase (PI3K)/AKT pathway (Zhu et al., 2019). Silencing MALAT1 suppresses colorectal cancer cell proliferation as well as expression levels of EZH2 by upregulating miR-363-3p (Xie et al., 2019). MALAT1 promotes stem cell-like phenotypes of pancreatic cancer cells *via* upregulating the expression of ZEB1 and self-renewal-related factor Sox2 (Jiao et al., 2015; Zhuo et al., 2018). These studies suggest that MALAT1 plays a crucial role in promoting cancer progression in pancreatic cancer. Besides, reportedly, p53 can exert its tumor-suppressive functions *via* repressing the transcription of MALAT1 (Wen et al., 2016; Fuschi et al., 2017). In the present work, it was confirmed that MALAT1 was positively regulated by IPO7 in pancreatic cancer cells. Thus, we concluded that the IPO7/p53/MALAT1 axis was involved in pancreatic cancer progression.

MiRNAs are also important regulators of malignant properties of cancer cells (Rupaimoole and Slack, 2017). Reportedly, miR-129-5p exerts anticancer effects in a variety of human tumors (Wang and Yu, 2018; Gao et al., 2019). Specifically, miR-129-5p restrains gastric cancer cell proliferation, migration, and invasion by suppressing COL1A1 (Wang and Yu, 2018). MiR-129-5p represses the growth of liver cancer cells *via* regulating calcium/calmodulin-dependent protein kinase 4 (CAMK4) and thus suppresses the activation of mitogen-activated protein kinase (MAPK) signaling (Lin et al., 2019). In glioblastoma, miR-129-5p represses Wnt5a expression and blocks the protein kinase C (PKC)/extracellular signal-regulated kinase (ERK)/nuclear factor (NF)-κB and c-Jun N-terminal kinase (JNK) pathways (Zeng et al., 2018). MiR-129-5p is also a tumor suppressor in pancreatic cancer, and it is reported that miR-129-5p exerts a tumor-suppressive effect in pancreatic cancer progression *via* targeting PBX3 (Qiu et al., 2019). In the present study, a miRNA response element for miR-129-5p on MALAT1 sequence was identified in pancreatic cancer cells, and it was revealed that miR-129-5p could be sponged and repressed by MALAT1. Importantly, IPO7 was validated as a novel target gene of miR-129-5p and could be negatively regulated by it. Considering that IPO7 could positively regulate MALAT1, therefore, the IPO7/p53/MALAT1/miR-129-5p positive feedback loop in pancreatic cancer progression was depicted. This positive feedback loop is helpful to explain the mechanism of sustaining the progression of pancreatic cancer and the mechanisms of p53 defect, MALAT1 overexpression, and miR-129-5p dysregulation in pancreatic cancer tissues.

Taken together, we demonstrate that IPO7 expression is up-modulated in pancreatic cancer tissues as well as cell lines, and its overexpression implies shorter survival time of the patients, suggesting that it may serve as a promising biomarker for evaluating the prognosis of patients with pancreatic cancer. Furthermore, gain-of-function and loss-of-function experiments suggested that IPO7/p53/MALAT1/miR-129-5p positive feedback loop promotes the malignant biological behaviors of pancreatic cancer cells, which provides useful clues for the diagnosis and treatment of pancreatic cancer Graphical Abstract.

## Data Availability Statement

The original contributions presented in the study are included in the article/Supplementary Material, further inquiries can be directed to the corresponding author/s.

## Ethics Statement

This research was endorsed by the Ethics Committee of Shengjing Hospital and performed according to the Declaration of Helsinki. The patients/participants provided their written informed consent to participate in this study. The animal experiments were endorsed by the Experimental Animal Ethics Committee of Shengjing Hospital.

## Author Contributions

JX, WX, and QS: conceived and designed the experiments. JX, YX, ZL, and QS: performed the experiments. JX and QS: statistical analysis. JX, WX, YX, ZL, QS, and CL: wrote the manuscript. All authors read and approved the final manuscript.

## Conflict of Interest

The authors declare that the research was conducted in the absence of any commercial or financial relationships that could be construed as a potential conflict of interest.

## Publisher’s Note

All claims expressed in this article are solely those of the authors and do not necessarily represent those of their affiliated organizations, or those of the publisher, the editors and the reviewers. Any product that may be evaluated in this article, or claim that may be made by its manufacturer, is not guaranteed or endorsed by the publisher.

## Footnotes

1
http://gepia2.cancer-pku.cn/#index

2
http://www.proteinatlas.org/

3
www.oncolnc.org

## Supplementary Material

Supplementary Figure 1 **(A,B)** qPCR **(A)** and Western blot **(B)** were used to detect the expression of IPO7 in pancreatic cancer cells after cell transfection. The results showed that the transfection reagent didn’t change the expression level of IPO7 in pancreatic cancer cells.

Supplementary Figure 2 The expression of MALAT1 in HAPC **(A)** and PANC-1 cell **(B)** were detected by qPCR, after p53, MALAT1, miR-129-5p, and IPO7 were selectively regulated.

Supplementary Figure 3 Three online databases, including miRDB, TargetScan, and StarBase were searched computationally for potential target genes that are complementary to miR-129-5p. Three hundred twenty-seven candidate targets were obtained including IPO7.

Supplementary Figure 4 The KEGG pathways enriched for the miR-129-5p targets were associated with multiple pathways, including the Hippo, WNT, and p53 signaling pathway, etc.
