# Supplementary figures and images for: Pancreatic Cancer Progression Is Regulated by IPO7/p53/LncRNA MALAT1/MiR-129-5p Positive Feedback Loop
Source: Front Cell Dev Biol. 2021 Oct 1;9:630262. doi: 10.3389/fcell.2021.630262 (PMC8517143; doi:10.3389/fcell.2021.630262)

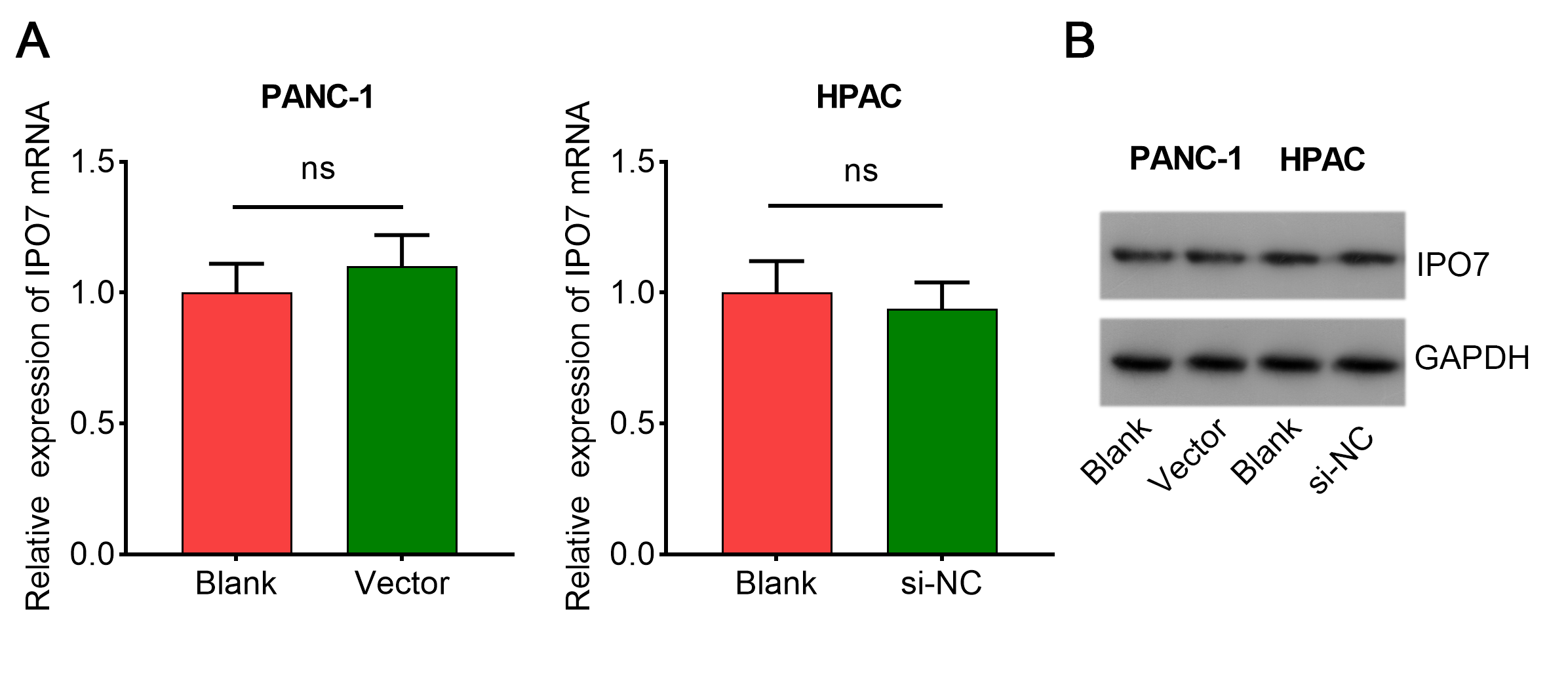

Supplement: Supplementary Figure 1 — (A,B) qPCR (A) and Western blot (B) were used to detect the expression of IPO7 in pancreatic cancer cells after cell transfection. The results showed that the transfection reagent didn’t change the expression level of IPO7 in pancreatic cancer cells. [file Image_1.tif]

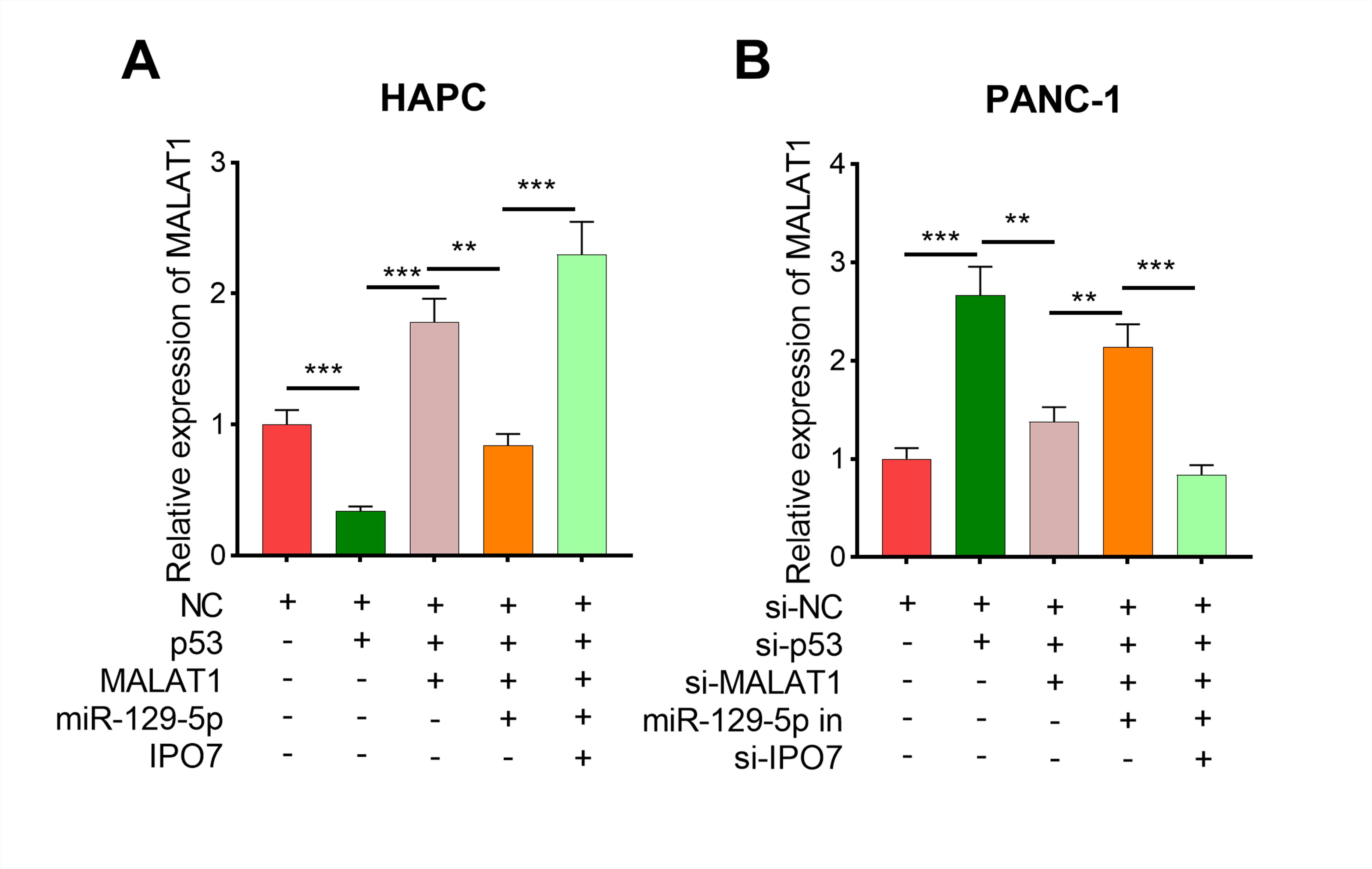

Supplement: Supplementary Figure 2 — The expression of MALAT1 in HAPC (A) and PANC-1 cell (B) were detected by qPCR, after p53, MALAT1, miR-129-5p, and IPO7 were selectively regulated. [file Image_2.tif]

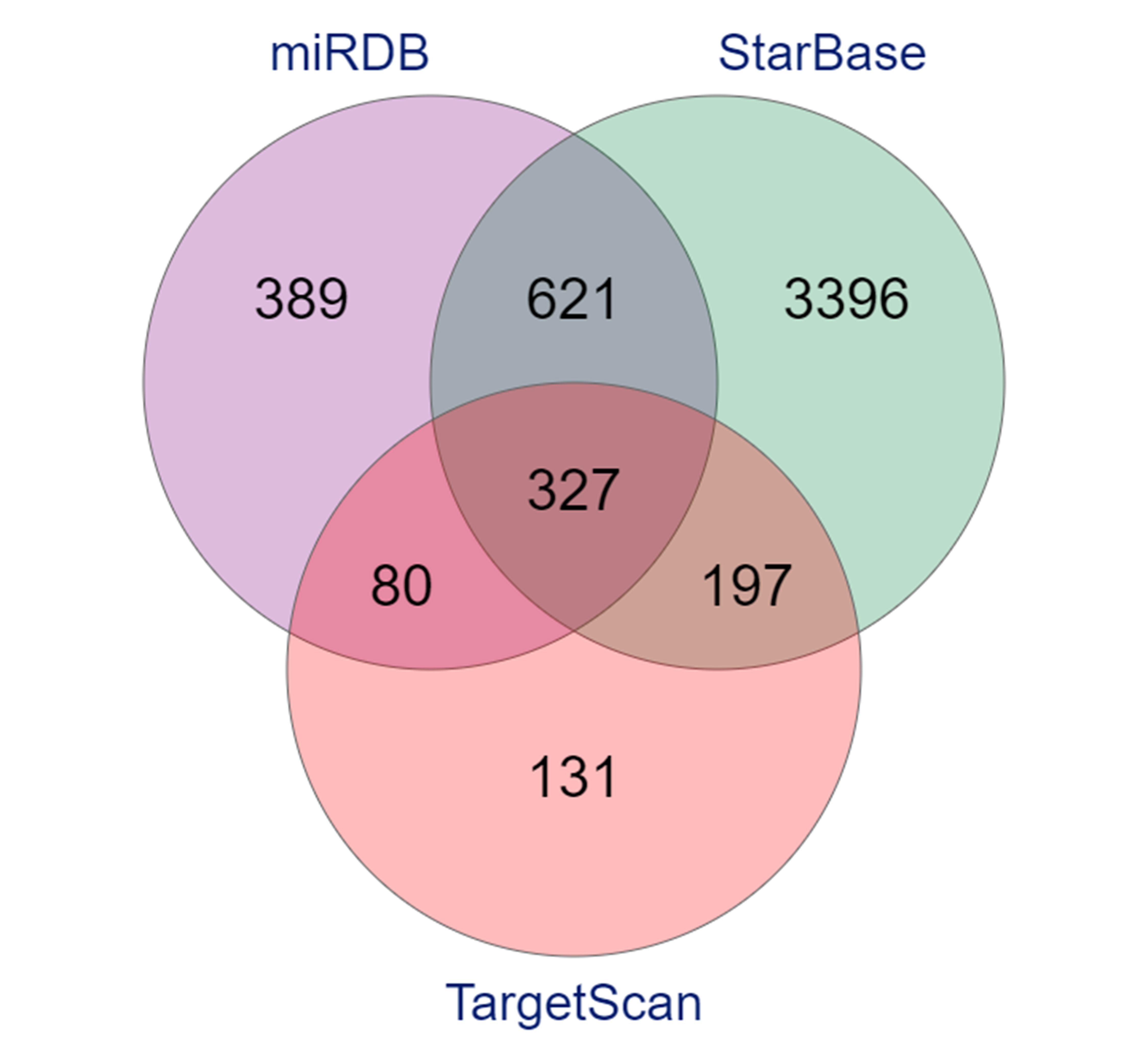

Supplement: Supplementary Figure 3 — Three online databases, including miRDB, TargetScan, and StarBase were searched computationally for potential target genes that are complementary to miR-129-5p. Three hundred twenty-seven candidate targets were obtained including IPO7. [file Image_3.tif]

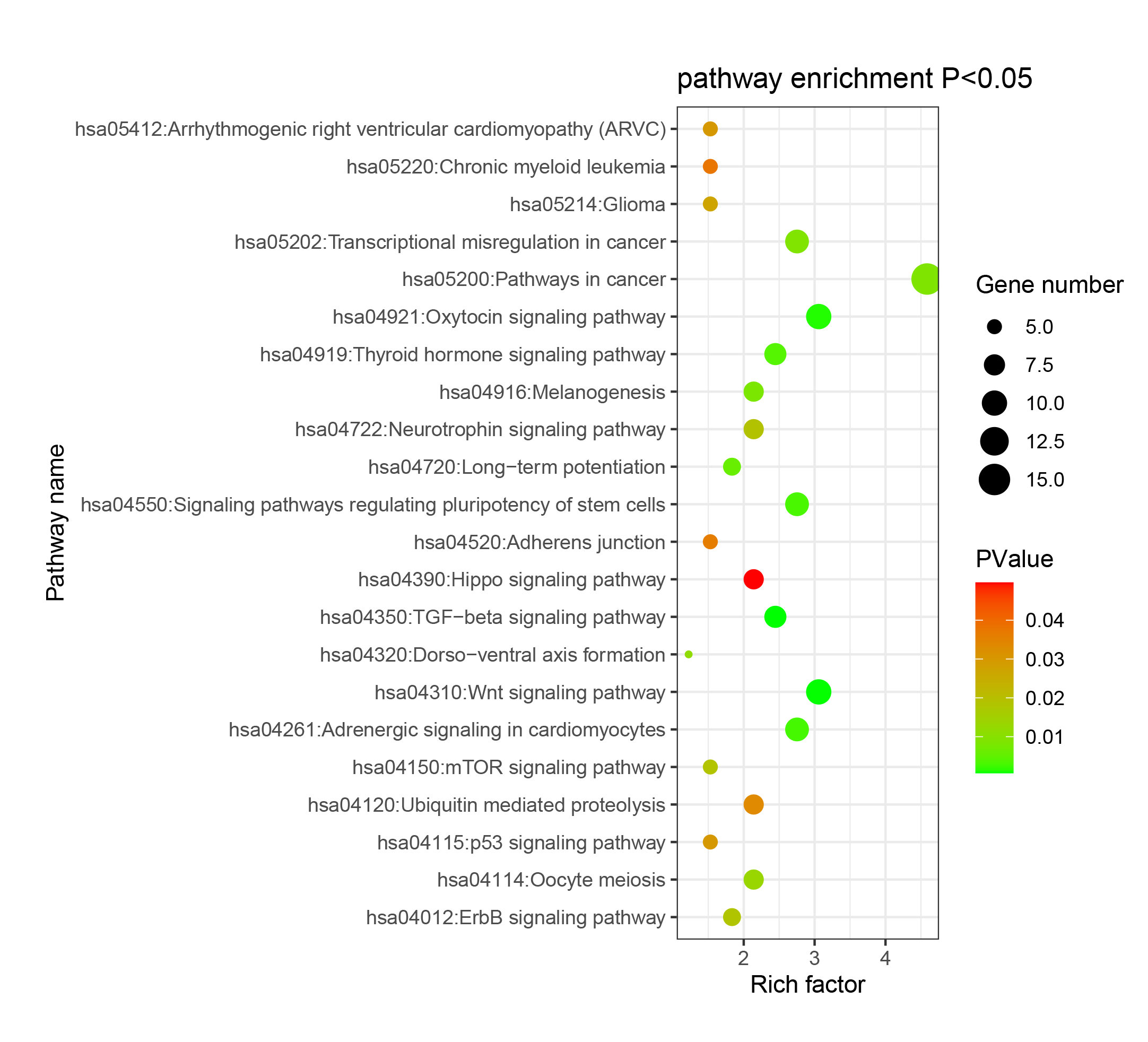

Supplement: Supplementary Figure 4 — The KEGG pathways enriched for the miR-129-5p targets were associated with multiple pathways, including the Hippo, WNT, and p53 signaling pathway, etc. [file Image_4.tif]
